# Endothelial Dysfunction and Impaired Wound Healing Following Radiation Combined Skin Wound Injury

**DOI:** 10.3390/ijms252312498

**Published:** 2024-11-21

**Authors:** Li Wang, Bin Lin, Min Zhai, Lisa Hull, Wanchang Cui, Mang Xiao

**Affiliations:** 1 Radiation Combined Injury Program, Armed Forces Radiobiology Research Institute, Uniformed Services University of the Health Sciences, Bethesda, MD 20814, USA; bin.lin.ctr@usuhs.edu (B.L.); min.zhai.ctr@usuhs.edu (M.Z.); lisa.hull.ctr@usuhs.edu (L.H.); wanchang.cui.ctr@usuhs.edu (W.C.); 2 Henry M Jackson Foundation for the Advancement of Military Medicine, Inc., Bethesda, MD 20817, USA; 3 Department of Pathology, Uniformed Services University of the Health Sciences, Bethesda, MD 20814, USA; 4 Department of Pharmacology and Molecular Therapeutics, Uniformed Services University of the Health Sciences, Bethesda, MD 20814, USA

**Keywords:** radiation combined skin wound injury, impaired wound healing, endothelial dysfunction, systemic and local proinflammatory response, TGFβ expression, granulation tissue formation, myofibroblast, collagen deposition

## Abstract

Currently, there are no U.S. Food and Drug Administration (FDA)-approved medical countermeasures (MCMs) for radiation combined injury (RCI), partially due to limited understanding of its mechanisms. Our previous research suggests that endothelial dysfunction may contribute to a poor prognosis of RCI. In this study, we demonstrated an increased risk of mortality, body weight loss, and delayed skin wound healing in RCI mice compared to mice with skin wounds alone or radiation injury (RI) 30 days post-insult. Furthermore, we evaluated biomarkers of endothelial dysfunction, inflammation, and impaired wound healing in mice at early time points after RCI. Mice were exposed to 9.0 Gy total-body irradiation (TBI) followed by skin wound. Samples were collected on days 3, 7, and 14 post-TBI. Endothelial dysfunction markers were measured by ELISA, and skin wound healing was assessed histologically. Our results show that endothelial damage and inflammation are more severe and persistent in the RCI compared to the wound-alone group. Additionally, RCI impairs granulation tissue formation, reduces myofibroblast presence, and delays collagen deposition, correlating with more severe endothelial damage. TGF signaling may play a key role in this impaired healing. These findings suggest that targeting the endothelial dysfunction and TGF-β pathways may provide potential therapeutic strategies for improving delayed wound healing in RCI, which could subsequently influence outcomes such as survival after RCI.

## 1. Introduction

Radiation combined injury (RCI) is a condition in which an individual is exposed to ionizing radiation along with another form of injury, such as physical trauma (e.g., thermal burns, wounds, or fracture), hemorrhage, or infection [1,2,3,4]. This condition is particularly concerning in situations like nuclear accidents or radiological disasters, where people may be exposed to radiation and also sustain injuries from blasts or fires [3,4]. Research indicates that around 60% of injuries in mass casualty incidents involving radiation are likely to be RCI [3]. The coexistence of these injuries complicates treatment and significantly worsens the patient’s prognosis [2,3,4]. Currently, there are no U.S. Food and Drug Administration (FDA)-approved treatments specifically for RCI.

Total-body irradiation (TBI) combined with skin wound trauma represents a specific type of RCI that significantly hinders recovery due to delayed wound closure [1,2,3,5], subsequently increasing the risk of infection [1,3,6]. Scientists at AFRRI have developed a RCI model using B6D2F1/J mice (TBI followed by skin wound), providing a valuable tool for studying radiation combined skin wound injury [1,6,7,8] and assessing potential MCMs [2,4,6,9,10,11]. B6D2F1/J mice, a hybrid of the C57BL/6J and DBA/2J strains, were selected for the RCI studies due to their hybrid vigor, which includes improved stress resilience and greater disease resistance [12,13]. Research at AFRRI indicates that RCI delays wound healing, leading to higher mortality rates compared to radiation injury (RI) alone by increasing susceptibility to infection [6,7].

Radiation exposure directly damages endothelial cells, causing cell death and impaired function [14]. Reduced nitric oxide (NO) production by injured endothelial cells causes blood vessel constriction, decreasing blood flow to irradiated tissue, which impairs oxygen delivery, slows healing, and may lead to organ failure [15]. During the skin wound healing process, granulation tissue, which is rich in blood vessels, immune cells, and fibroblasts, is essential for wound closure [16]. Endothelial dysfunction disrupts the angiogenesis required for granulation tissue formation, thereby slowing wound closure [17]. These findings suggest that endothelial dysfunction is a key factor in both radiation injury and skin wounds individually.

In our previously study using a mouse model of RCI, we found that the oral administration of L-citrulline at 1 g/kg once daily for 21 days, starting 24 h post-TBI, increased 30-day survival by 25% and significantly accelerated wound healing after 9.5 Gy RCI [2]. Endothelial dysfunction, characterized by impaired production or bioavailability of NO [18,19], may be addressed by L-citrulline treatment, which enhances NO production [20]. L-citrulline is converted to L-arginine in the kidneys and intestines, and L-arginine serves as a precursor for NO production by endothelial cells. This study suggested that endothelial dysfunction may play a role in RCI, particularly in the delayed wound healing observed following RCI. Furthermore, we demonstrated that RCI induces persistent proinflammatory responses, both systemically and locally [1]. Since inflammation can trigger endothelial dysfunction [21], our findings suggested that endothelial dysfunction may contribute to the effects of RCI. However, direct evidence linking endothelial dysfunction to the RCI-induced delay in wound healing remains elusive [22]. In this study, using the same mouse model of RCI, we measured the biomarkers of endothelial dysfunction and inflammation in both serum and skin wounds on days 3, 7, and 14 after 9.0 Gy RCI. Days 3, 7, and 14 post-RCI were selected to capture the early, peak, and late phases of the acute inflammatory response and/or endothelial damage, respectively [23]. We also assessed the impaired wound healing at these time points.

## 2. Results

### 2.1. RCI Resulted in Higher Mortality and Lower Body Weight than RI and Slowed Down Skin Wound Healing

**Figure 1:**
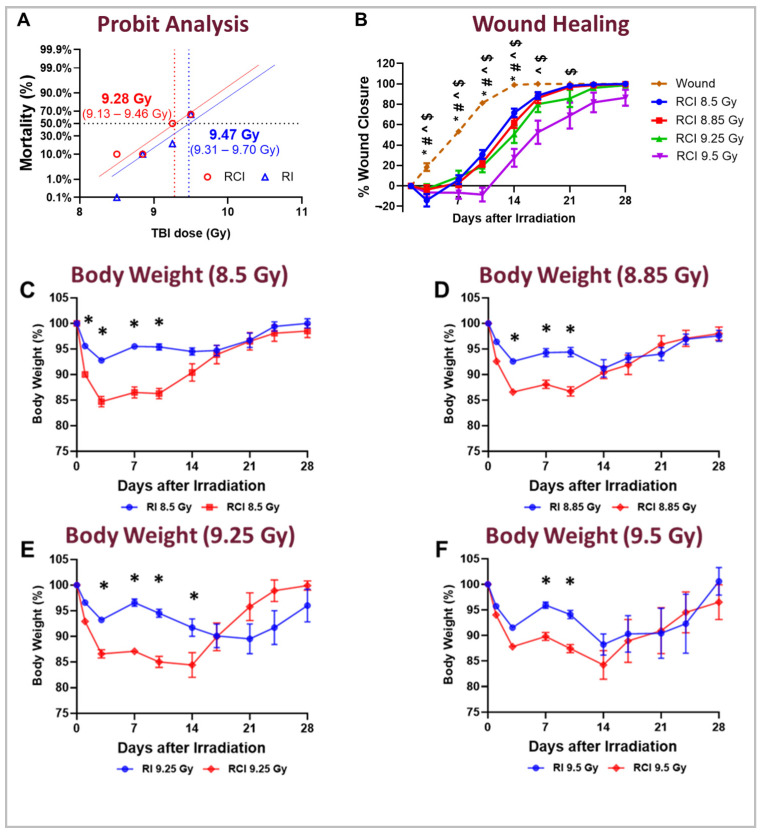
Effect of different radiation doses on mouse survival, wound healing, and body weight loss following skin wound alone, RI, and RCI. Mice in both the RI and RCI groups were exposed to varying radiation doses: 8.5 Gy, 8.85 Gy, 9.25 Gy, and 9.5 Gy. (**Panel A**) Radiation dose–response curves for RI mice (blue) and RCI mice (red) are shown. The LD_50/30_ (the radiation dose expected to cause lethality to 50% of an exposed population within 30 days) was determined using a probit analysis. For RI mice, the LD_50/30_ is 9.47 Gy (95% confidence interval: 9.31–9.70 Gy), while for RCI mice, the LD_50/30_ is 9.28 Gy (95% confidence interval: 9.13–9.46 Gy). (**Panel B**) The delay in skin wound healing was observed in a dose-dependent manner: wounds alone (dashed line, bronze); 8.5 Gy RCI (solid line, blue); 8.85 Gy RCI (solid line, red); 9.25 Gy RCI (solid line, green); and 9.5 Gy RCI (solid line, purple). Significant differences in wound healing were observed: * *p* < 0.05 for the wound-alone group vs. 8.5 Gy RCI; # *p* < 0.05 for the wound-alone group vs. 8.85 Gy RCI; ^ *p* < 0.05 for the wound-alone group vs. 9.25 Gy RCI; $ *p* < 0.05 for the wound-alone group vs. 9.5 Gy RCI, determined by two-way ANOVA followed by Dunnett’s multiple comparison test. The sample size ranged from N = 7 to 20 per group per time point. (**Panel C**–**F**) RCI mice showed more body weight loss compared to RI mice across all tested doses: 8.5 Gy, 8.85 Gy, 9.25 Gy, and 9.5 Gy (RI: blue; RCI: red). Significant differences in weight loss were observed (* *p* < 0.05 for RI vs. RCI), determined by two-way ANOVA followed by Sidak’s multiple comparison test. The sample size ranged from N = 7 to 20 per group per time point. Data in panel B to panel F are expressed as mean ± standard error of the mean (SEM).

The mortality rates of RCI (ionizing radiation combined with skin wound injury) and RI (radiation injury alone) were compared. Mice in the RCI and RI groups were exposed to different doses of radiation (8.5, 8.85, 9.25, and 9.5 Gy; n = 20 per group per radiation dose), and 30-day survival, body weight, and wound size were monitored. For the RCI mice, the survival rates at day 30 were determined, and the lethal radiation doses (LD) resulting in 10%, 30%, 50%, 70%, and 90% mortality (LD_10/30_, LD_30/30_, LD_50/30_, LD_70/30_, and LD_90/30_) were calculated as 8.71 Gy, 9.04 Gy, 9.28 Gy, 9.53 Gy, and 9.89 Gy, respectively. For the RI mice, the LD_10/30_, LD_30/30_, LD_50/30_, LD_70/30_, and LD_90/30_ were determined as 8.89 Gy, 9.23 Gy, 9.47 Gy, 9.72 Gy, and 10.10 Gy, respectively. Overall, these results indicate that RCI resulted in higher mortality rates compared to RI, as evidenced by a lower LD_50/30_ value of 9.28 Gy for the RCI mice compared to 9.47 Gy for the RI mice (Figure 1A). We also observed that, compared to the wound-alone animals, the RCI mice displayed a significant, dose-dependent delay in wound healing (Figure 1B). In addition, weight loss in the RCI mice was markedly greater than in the RI mice from day 3 to day 10 post-TBI at each dose tested (Figure 1C–F).

### 2.2. The Proinflammatory Factors and Endothelial Damage Markers Were Significantly Increased and Persistent in RCI Mice Compared to Wound-Alone Mice

**Figure 2:**
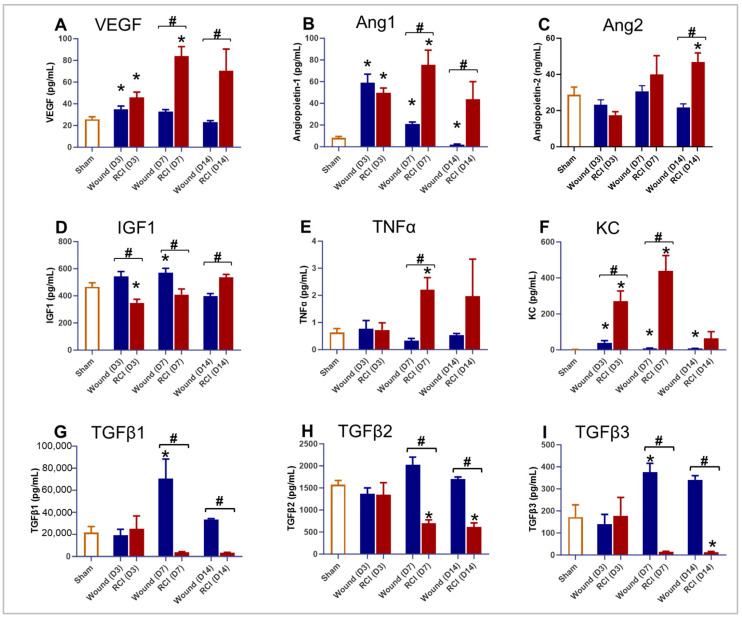
Effect of RCI on serum markers associated with endothelial dysfunction and inflammation. Mice were subjected to sham injury, wounds alone, and 9.0 Gy RCI, and sera were collected on days 3, 7, and 14 post-injury. Various markers associated with endothelial dysfunction and inflammation were assessed using ELISA. The markers analyzed include (**Panel A**) vascular endothelial growth factor (VEGF); (**Panel B**) Angiopoietin-1 (Ang1); (**Panel C**) Angiopoietin-2 (Ang2); (**Panel D**) insulin-like growth factor 1 (IGF1); (**Panel E**) tumor necrosis factor alpha (TNFα); (**Panel F**) keratinocyte-derived cytokine (KC, also known as CXCL-1); (**Panel G**) transforming growth factor-beta 1 (TGFβ1); (**Panel H**) TGFβ2; and (**Panel I**) TGFβ3. Data are expressed as mean ± SEM. Significant differences in the mentioned markers were observed: * *p* < 0.05 for Sham vs. Wound/RCI on days 3, 7, and 14 post-injury, determined by one-way ANOVA followed by Dunnett’s multiple comparison test; # *p* < 0.05 for Wound vs. RCI at the same time point, determined by two-way ANOVA followed by Sidak’s multiple comparison test. The sample size ranged from N = 3 to 9 per group per time point.

The exacerbated combined effects of RCI may be attributed to the amplification of shared pathophysiological pathways, such as endothelial dysfunction and inflammation, which are commonly impacted by both RI [14,24,25,26,27,28] and skin wound trauma [29,30,31]. To investigate the endothelial damage induced by RCI, we evaluated the serum proinflammatory cytokines and factors associated with endothelial dysfunction at three time points: days 3, 7, and 14 post-injury. Compared to the sham group, the wound-alone and/or RCI groups significantly increased the amount of vascular endothelial growth factor (VEGF, also known as vascular permeability factor, ‘VPF’), Angiopoietin-1 (Ang1), Angiopoietin-2 (Ang2), insulin-like growth factor 1 (IGF1), tumor necrosis factor alpha (TNFα), and keratinocyte-derived cytokine (KC, also known as CXCL-1) in circulation (Figure 2A–F). More importantly, RCI significantly increased the levels and/or prolonged the persistence of serum VEGF, Ang1, Ang2, TNFα, and KC compared to the wound-alone group (Figure 2A–C,E,F). Furthermore, compared to the wound-alone groups, the serum IGF1 levels were significantly reduced on days 3 and 7 post-RCI, with levels returning to normal by day 14 post-RCI, as shown in Figure 2D. Transforming growth factor-beta 1 (TGFβ1), TGFβ2, and TGFβ3 are primarily anti-inflammatory. In comparison to the sham group, the wound-alone group significantly elevated TGFβ1 and TGFβ3 levels, while the RCI group showed markedly decreased TGFβ2 and TGFβ3 (Figure 2G–I). Notably, the RCI group had a significant reduction in TGFβ1, TGFβ2, and TGFβ3 levels compared to the wound-alone group (Figure 2G–I). These findings collectively suggest that RCI exacerbates endothelial damage and the inflammatory response.

### 2.3. RCI Hindered Granulation Tissue Formation Compared to Wounds Alone

**Figure 3:**
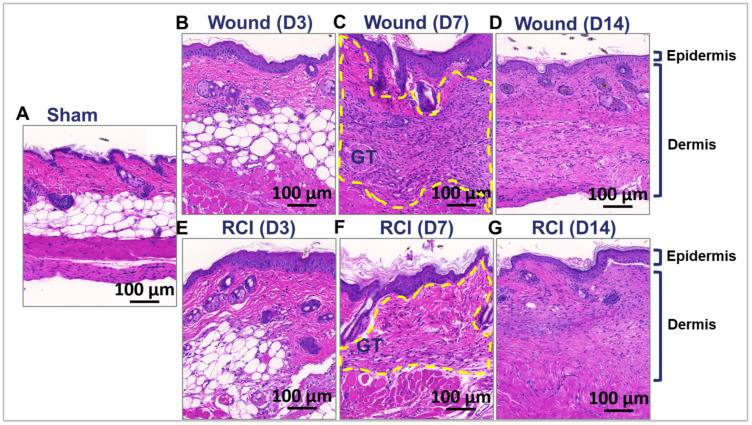
Effect of RCI on granulation tissue formation during skin wound healing. Mice were subjected to sham injury (**Panel A**), wounds alone (**Panel B**–**D**), and 9.0 Gy RCI (**Panel E**–**G**). Skin tissues surrounding the wounds were collected on days 3, 7, and 14 post-injury. Hematoxylin and eosin (H&E) staining was performed on formalin-fixed and paraffin-embedded skin cross sections, showing nuclei in blue-purple and cytoplasm in pink-red. Representative images of the skin peri-wound area display two main layers: the epidermis and the dermis. Granulation tissue formed on day 7 post-injury is outlined with a dotted yellow line. The scale bar represents 100 μm.

We further analyzed the skin wounds in the wound-alone and RCI groups. The gross morphology of wounds on days 3, 7, and 14 post-injury were examined using hematoxylin and eosin (H&E) staining, and the image data from representative mouse samples in different groups are shown in Figure 3. Compared to the dense connective and orderly organized tissue observed in normal skin from the sham group (Figure 3A), the granulation tissue in the wound-alone and RCI groups appeared loosely and irregular packed, indicating the active and dynamic process of tissue repair and new tissue formation. In the wound-alone group, the granulation tissue began to form in the peri-wound area during the proliferative phase, typically starting around day 3 (Figure 3B), progressing by day 7 (Figure 3C), and maturing by day 14 (Figure 3D). In contrast, granulation tissue formation in the RCI group was delayed (Figure 3E–G) and appeared reduced in size, particularly at the day 7 time point (Figure 3F).

### 2.4. RCI Did Not Affect the Infiltration of Neutrophils and Macrophages to the Skin Wounds Compared to Wounds Alone

**Figure 4:**
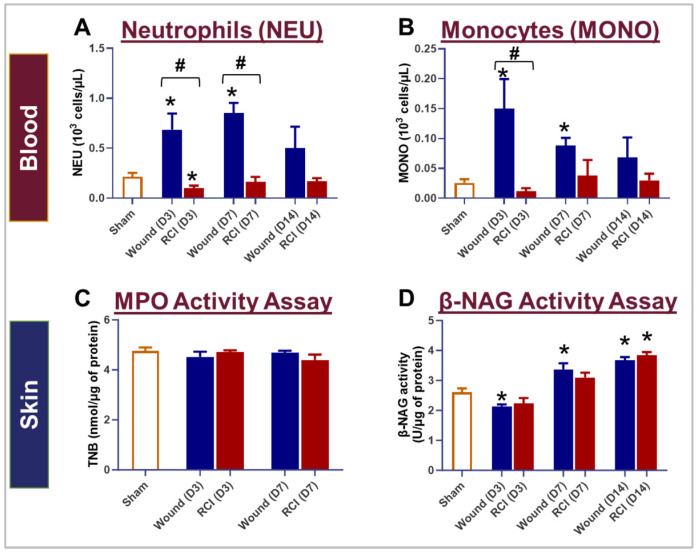
Impact of RCI on peripheral blood neutrophil and monocyte counts, and neutrophil and macrophage infiltration in skin wounds. Mice were subjected to sham injury, wounds alone, and 9.0 Gy RCI. Blood and skin tissues surrounding the wounds were collected on days 3, 7, and 14 post-injury. Complete blood count (CBC) with a differential analysis was performed on EDTA-treated blood to evaluate the types of blood cells, specifically (**Panel A**) neutrophils (NEU) and (**Panel B**) monocytes (MONO). To assess NEU and macrophage (Mϕ) infiltration in the wounds, the activity of (**Panel C**) Myeloperoxidase (MPO) and (**Panel D**) β-N-Acetylglucosaminidase (β-NAG) were measured in skin tissue lysates. Data are expressed as mean ± SEM. Significant differences were observed, indicated as * *p* < 0.05 for Sham vs. Wound/RCI on days 3, 7, and 14 post-injury, determined by one-way ANOVA followed by Dunnett’s multiple comparison test, and # *p* < 0.05 for Wound vs. RCI at the same time point, determined by two-way ANOVA followed by Sidak’s multiple comparison test. The sample size ranged from N = 4 to 9 per group per time point.

Compared to the sham group, skin wound trauma markedly increased neutrophil (NEU) and monocyte (MONO) counts in the peripheral blood on days 3 and 7 after injury, but this effect was abolished by RCI (Figure 4A,B). RCI resulted in a significant decrease in circulating NEU on day 3 compared to the sham group (Figure 4A), and significantly lower NEU and MONO counts on day 3 and/or day 7 compared to the wound-alone group (Figure 4A,B). To determine whether decreased NEU and MONO counts in the blood affected the immune cell infiltration in skin wound, we measured the activity of two enzymes, Myeloperoxidase (MPO) and β-N-Acetylglucosaminidase (β-NAG), in the wounded skin area. MPO and β-NAG serve as indirect markers for NEU and macrophage (Mϕ) infiltration into skin wounds. MONO in the bloodstream acts as a precursor to tissue Mϕ. No differences in MPO activity were observed among the sham, wound-alone, and RCI groups on days 3 and 7 post-injury (Figure 4C). However, the wound-alone and/or the RCI groups showed increased β-NAG activity on days 7 and 14 post-injury compared to the sham group (Figure 4D). More importantly, no significant differences in MPO and β-NAG activity were observed between the wound-alone and RCI groups (Figure 4C,D). This indicates that although RCI led to a significant decrease in circulating NEU and MONO compared to wounds alone, it did not affect the infiltration of NEU and Mϕ into the wounds.

### 2.5. RCI Induced an Insufficient Endothelial Response in Skin Wounds Compared to Wounds Alone

**Figure 5:**
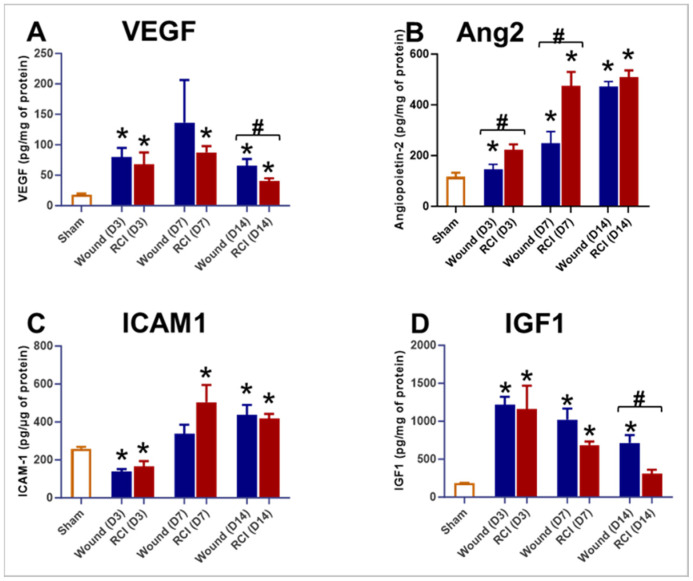
Effect of RCI on skin tissue endothelial dysfunction markers. Mice were subjected to sham injury, wounds alone, and 9.0 Gy RCI, and skin tissues surrounding the wounds were collected on days 3, 7, and 14 post-injury. Various markers associated with endothelial dysfunction were assessed in skin tissue lysates using ELISA. The markers analyzed include (**Panel A**) vascular endothelial growth factor (VEGF); (**Panel B**) Angiopoietin-2 (Ang2); (**Panel C**) intercellular adhesion molecule 1 (ICAM1); and (**Panel D**) insulin-like growth factor 1 (IGF1). Data are expressed as mean ± SEM. Significant differences in these markers were observed: * *p* < 0.05 for Sham vs. Wound/RCI on days 3, 7, and 14 post-injury, determined by one-way ANOVA followed by Dunnett’s multiple comparison test, and # *p* < 0.05 for Wound vs. RCI at the same time point, determined by two-way ANOVA followed by Sidak’s multiple comparison test. The sample size ranged from N = 3 to 9 per group per time point.

Compared to the sham group, both the wound-alone and RCI groups showed significantly elevated levels of VEGF, Ang2, ICAM1, and IGF1 (Figure 5A–D). However, compared to the wound-alone group, the RCI mice displayed significant lower levels of VEGF and IGF1 on day 14 post-injury (Figure 5A–D) and markedly higher levels of Ang2 on days 3 and 7 post-injury (Figure 5B). This suggests that RCI led to an impaired endothelial response in skin wounds compared to wounds alone.

### 2.6. Altered TGFβ Signaling in the Skin of RCI Mice Compared to the Wound-Alone Group

**Figure 6:**
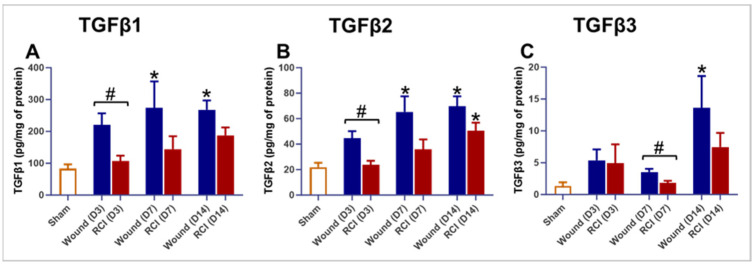
Effect of RCI on skin tissue levels of transforming growth factor-beta (TGFβ) 1-3. Mice were subjected to sham injury, wounds alone, and 9.0 Gy RCI, and skin tissues surrounding the wounds were collected on days 3, 7, and 14 post-injury. Skin tissue levels of (**Panel A**) TGFβ1, (**Panel B**) TGFβ2, and (**Panel C**) TGFβ3 were measured using the Bio-Plex multiplexing system. Data are expressed as mean ± SEM. Significant differences in TGFβ1-3 were observed: * *p* < 0.05 for Sham vs. Wound/RCI on days 3, 7, and 14 post-injury, determined by one-way ANOVA followed by Dunnett’s multiple comparison test, and # *p* < 0.05 for Wound vs. RCI at the same time point, determined by two-way ANOVA followed by Sidak’s multiple comparison test. The sample size was N = 4 per group per time point.

To assess the impact of RCI on TGFβ expression in skin wounds, we measured the levels of TGFβ1, TGFβ2, and TGFβ3 on days 3, 7, and 14 post-injury in the skin tissues. Compared to the sham group, mice in the wound-alone group exhibited significantly higher levels of TGFβ1, TGFβ2, and TGFβ3 at multiple time points (Figure 6A–C). In contrast, the RCI mice showed significantly elevated TGFβ2 levels only on day 14 post-injury at the wound site (Figure 6B). More importantly, compared to the wound-alone group, RCI led to a significant reduction in TGFβ1 and TGFβ2 on day 3 post-injury and TGFβ3 on day 7 post-injury (Figure 6A–C). These findings suggest that the TGFβ expression is dysregulated in the skin of RCI mice compared to the wound-alone mice.

### 2.7. RCI Reduced CTGF but Not Fibronectin Levels in Skin Wounds Compared to Wounds Alone

**Figure 7:**
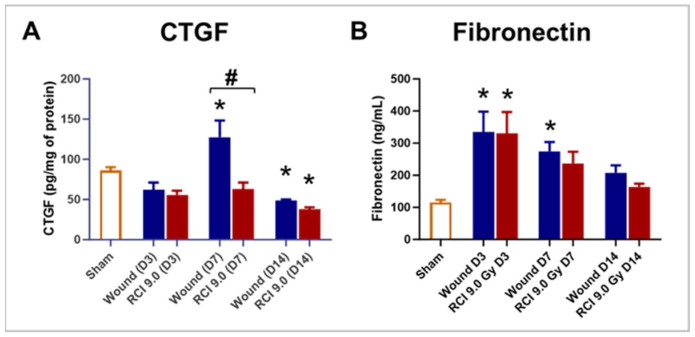
Effect of RCI on skin tissue levels of connective tissue growth factor (CTGF) and fibronectin. Mice were subjected to sham injury, wounds alone, and 9.0 Gy RCI, and skin tissues surrounding the wounds were collected on days 3, 7, and 14 post-injury. Levels of (**Panel A**) CTGF and (**Panel B**) fibronectin were measured in tissue lysates by EKISA. Data are expressed as mean ± SEM. Significant differences in these factors were observed: * *p* < 0.05 for Sham vs. Wound/RCI on days 3, 7, and 14 post-injury, determined by one-way ANOVA followed by Dunnett’s multiple comparison test, and # *p* < 0.05 for Wound vs. RCI at the same time point, determined by two-way ANOVA followed by Sidak’s multiple comparison test. The sample size ranged from N = 4 to 9 per group per time point.

To examine the effect of RCI on connective tissue growth factor (CTGF) expression in skin wounds, we measured its levels in skin wound tissues on days 3, 7, and 14 post-injury. Compared to the sham group, the wound-alone group showed a significant elevation in CTGF level on day 7 post-injury, an effect that was abolished by RCI (Figure 7A). More importantly, RCI significantly reduced CTGF levels on day 7 compared to the wound-alone group (Figure 7A). By day 14, CTGF levels in both the wound-alone and the RCI groups had declined to levels even lower than those in the sham group (Figure 7A). Granulation tissue contains a provisional extracellular matrix (ECM) rich in fibronectin, collagen, and glycosaminoglycans [16]. In fibrotic conditions, CTGF has been shown to upregulate fibronectin expression [32]. This prompted us to evaluate fibronectin levels in skin wounds at the same time points. Compared to the sham group, fibronectin levels were significantly elevated in the wound-alone group on days 3 and 7 post-injury, and in the RCI group on day 3 (Figure 7B). However, no differences in fibronectin levels were observed between the wound-alone and the RCI groups (Figure 7B).

### 2.8. RCI Reduced Myofibroblast Presence and Delays Collagen Deposition in Skin Wounds Compared to Wounds Alone

**Figure 8:**
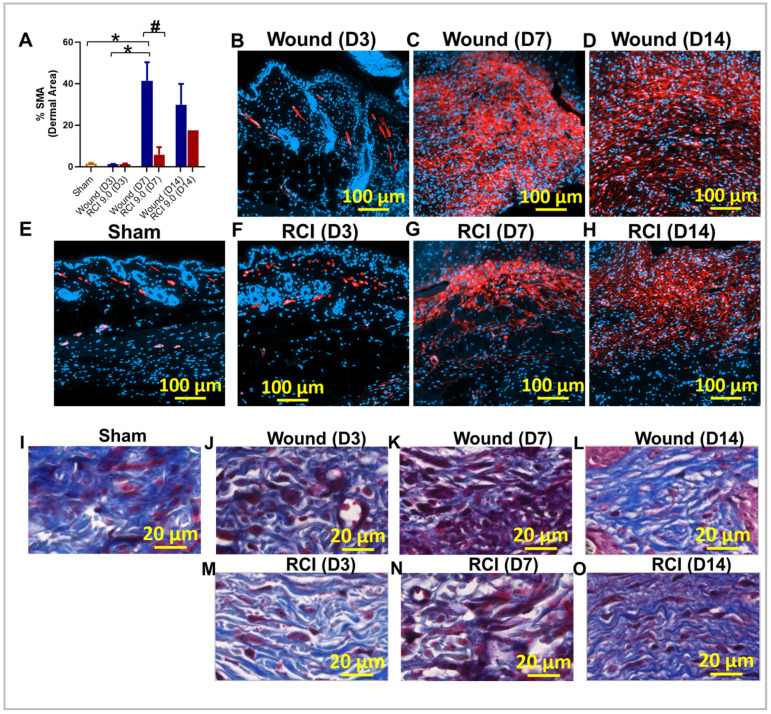
Effect of RCI on fibroblast differentiation and collagen deposition during skin wound healing. Mice were subjected to sham injury (**Panel E**,**I**), wounds alone (**Panel B**–**D**,**J**–**L**), and 9.0 Gy RCI (**Panel F**–**H**,**M**–**O**). Skin tissues surrounding the wounds were collected on days 3, 7, and 14 post-injury. (**Panel A**–**H**) Immunohistochemical (IHC) staining using anti-alpha-smooth muscle actin (anti-α-SMA) antibody was performed on formalin-fixed and paraffin-embedded skin cross sections, highlighting α-SMA in red and nuclei in blue. (**Panel A**) Quantitative analysis of the percentage of α-SMA (% SMA) signal in the dermal area is shown. Data are expressed as mean ± SEM. Significant differences in % SMA were observed: * *p* < 0.05 for difference among Sham, Wound, and RCI on days 3, 7, and 14 post-injury, determined by one-way ANOVA followed by Tukey’s multiple comparison test, and # *p* < 0.05 for Wound vs. RCI at the same time point, determined by two-way ANOVA followed by Sidak’s multiple comparison test. The sample size ranged from N = 3 to 4 per group per time point (except for N = 1 for RCI at the day 14 post-injury time point). The scale bar represents 100 μm. (**Panel I**–**O**) Masson’s trichrome staining was performed on formalin-fixed and paraffin-embedded skin cross sections, showing collagen fibers in blue, nuclei in black, and cytoplasm in red. The scale bar represents 20 μm.

To assess RCI’s effects on fibroblast differentiation during skin wound healing, we measured myofibroblast activity using immunohistochemistry (IHC) staining for its marker alpha-smooth muscle actin (α-SMA) and the ECM protein collagen. Compared to the sham group (Figure 8E), the wound-alone group showed a significant upregulation of α-SMA expression on day 7 post-injury (Figure 8A,C); however, this effect was abolished by RCI (Figure 8A,G). More importantly, the RCI mice exhibited a significant reduction in α-SMA expression compared to the wound-alone group on day 7 (Figure 8A,C,G). By day 14, the difference in α-SMA expression between the wound-alone and the RCI groups was no longer significant (Figure 8A,D,H). We also performed Masson’s trichrome staining to visualize collagens in the wounds. In the sham group, densely packed, well-organized collagen fibers were present throughout the dermis (Figure 8I). In the wound-alone group, Masson’s trichrome staining revealed (1) on day 3 post-injury, a loose, disorganized matrix with sparse collagen fibers in the wound area (Figure 8J); (2) on day 7, more prominent collagen fibers with a high density of red-stained inflammatory cells (Figure 8K); and (3) on day 14, collagen fibers that were packed and aligned parallel to the wound surface (Figure 8L). In contrast, RCI delayed collagen deposition and resulted in sparse, disorganized collagen fibers and prolonged inflammatory cell infiltration (Figure 8M–O).

## 3. Discussion

Endothelial dysfunction, characterized by impaired vascular function, has emerged as a key factor in various pathophysiological processes [33], including delayed wound healing [34]. Healthy endothelial function and a well-regulated inflammatory response are essential for wound healing [35,36,37]. Endothelial function is closely linked to granulation tissue formation, which relies on key components such as growth factors and ECM [16,38]. CTGF can be induced by TGFβ and plays a critical role in coordinating ECM production during wound healing [39]. It is essential for the differentiation of fibroblasts into myofibroblasts, acting either directly or as a mediator in the TGFβ signaling pathway [40,41]. Myofibroblasts are the primary source of collage during wound healing [42]. This study investigated the complex relationship between endothelial dysfunction and delayed wound healing in the context of ionizing radiation combined with skin wound injury (RCI), with a focus on the underlying mechanisms. The findings provide direct evidence of more persistent and severe endothelial damage and inflammation, both systemically and locally, in RCI, leading to delayed wound healing in a radiation dose-dependent fashion, as shown in Figure 1B. In addition, the results suggest that, unlike skin wounds alone, RCI impairs granulation tissue formation, reduces myofibroblast presence, and delays collagen deposition, which corresponds with more severe endothelial damage in skin wounds. These findings support our hypothesis that RCI triggers endothelial dysfunction, contributing to delayed wound healing. The lack of FDA-approved MCMs for preventing, mitigating, or treating RCI is primarily due to a limited understanding of the mechanisms underlying its harmful effects. This study provides proof of principle that targeting endothelial dysfunction may be a viable strategy for preventing, mitigating, or treating RCI.

TGF-β signaling plays a critical role in regulating endothelial function [43], suppressing inflammation [44], and promoting ECM deposition [45], all of which are essential for proper wound healing. A delicate balance of TGFβ signaling is essential for optimal wound repair. Excessive TGFβ signaling can lead to fibrosis, while insufficient signaling may result in delayed healing and chronic wounds [46,47]. TGFβ also plays a key role in regulating the endothelial response during wound healing [46]. Disruption of TGF-β signaling can result in endothelial dysfunction, impaired angiogenesis, and abnormal ECM remodeling, all contributing to delaying wound healing [48]. In this study, we found that, compared to wounds alone, RCI suppressed TGF-β expression both systemically and locally during the inflammatory and proliferative phases of wound healing. This dysregulation of TGF-β expression in RCI resulted in reduced ECM production in skin wounds. Furthermore, RCI decreased VEGF and increased Ang2 in mouse skin samples (Figure 5A,B). VEGF is a key factor in angiogenesis, necessary for delivering oxygen and nutrients to the wound site [38]; a reduction in VEGF levels could result in inadequate blood supply to the wound, delaying granulation tissue formation, which is crucial for wound healing. Moreover, elevated Ang2 levels could destabilize blood vessels, making them more prone to leakage [49]. Although Ang2 is involved in angiogenesis, excessive levels can disrupt the process, leading to impaired blood vessel formation [49].

Granulation tissue contains immune cells such as Mϕ, lymphocytes, and sometimes NEU, which help clear debris, fight infection, and release growth factors and cytokines [16,50,51]. Ionizing radiation damages bone marrow (BM), leading to decreased production of all types of blood cells [52]. Previous studies have revealed that the systemic factors, such as TBI-induced hematopoietic suppression, impair wound healing by significantly reducing immune cell infiltration in skin wounds [53]. Our group recently published a study demonstrating that delayed wound closure is not solely dependent on the extent of bone marrow damage but is significantly influenced by systemic inflammation and local factors induced by radiation [1]. That finding was based on data collected from animals surviving 30 days after TBI [1]. In the current study, we gathered data from earlier time points—days 3, 7, and 14 post-TBI—and identified that, in addition to systemic inflammation, both systemic and local endothelial dysfunction influence wound healing after RCI. Furthermore, we provided compelling evidence that, although RCI significantly reduced NEU and MONO levels in circulation compared to the wound-alone group, it did not affect the infiltration of NEU and Mϕ into the wounds.

While this study provides strong evidence linking the RCI to endothelial dysfunction and delayed wound healing, it is important to acknowledge that we did not conduct a comprehensive functional assessment of the endothelium and vasculature. Future studies could address this limitation by employing techniques like intravital microscopy or laser Doppler flowmetry to evaluate blood flow in the microcirculation [54], which is critical for wound healing. Incorporating these functional assessments would provide a more complete understanding of the mechanisms underlying endothelial dysfunction and delayed wound healing in RCI.

In the study, we estimated the LD_50/30_ to be 9.47 Gy (95% confidence interval: 9.31–9.70 Gy) for RI mice and 9.28 Gy (95% confidence interval: 9.13–9.46 Gy) for RCI mice. These results align with a previous AFRRI study, which reported an LD_50/30_ of 9.65 Gy (95% confidence interval: 9.51–9.82) for RI and 8.95 Gy (95% confidence interval: 8.74–9.11) for RCI [7]. Our findings further confirm that impaired wound healing and significant weight loss are characteristic features of the RCI mouse model, consistent with previous research [1,2]. The alignment between our results and the historical data from AFRRI further validates the reliability of this mouse model for establishing preclinical biomarkers and evaluating MCMs for RCI—an essential step, given the ethical and practical constraints of human studies.

Delayed skin wound healing in RCI can significantly affect outcomes, including survival [1,2]. Improperly healed skin wounds, as observed in RCI mice, are at a higher risk of infection and can lead to increased mortality [1,2]. By addressing this issue and investigating the role of TGF-β, this study may help identify potential therapeutic targets for treating delayed wound healing in RCI. In our future study, we will further explore the role of TGF-β signaling in RCI-induced delay in skin wound healing.

## 4. Materials and Methods

### 4.1. Mice and Ethics Statement

Female B6D2F1 mice (10 weeks old) were purchased from Jackson Laboratories (Bar Harbor, ME). Due to the aggressive behavior of the wounded male mice toward cage mates, which can lead to additional injuries, male mice were not tested. The mice were housed, up to five per cage, in a controlled environment with a 12:12 h light–dark cycle, room temperature of 23 ± 3 °C, and humidity of 50 ± 20% within a facility accredited by the Association for Assessment and Accreditation of Laboratory Animal Care, International (AAALAC International) at the Uniformed Services University of the Health Sciences (USUHS) [1,2]. Acidified water (pH 2.5–3.0) and Teklad global rodent diet 8604 (Inotiv, West Lafayette, IN, USA) were provided ad libitum. The mice were acclimated for at least two weeks prior to initiation of the experiments [1,2]. All animal manipulations were conducted in accordance with the USUHS guidelines and regulations as approved by the Institutional Animal Care and Use Committee (IACUC). USUHS IACUC Policy: Establishment of Humane Endpoints was implemented for judging morbidity and moribundity, especially during the critical period where increased morbidity and mortality are expected. During the critical period, the morbid mice were examined at least three times daily—early morning, afternoon, and evening—by the staff from the principal investigator group and the Department of Laboratory Animal Resources (DLAR), including weekends and holidays. The mice were considered moribund based on the following criteria: (1) blue mucus membranes/skin; (2) abdominal breathing (±gasping or open mouth breathing); (3) inability to stand; (4) not righting themselves when placed on their side within 5 s; (5) 35% or more body weight loss; and (6) scored at 12 or more. Moribund animals were euthanized following the American Veterinary Medical Association (AVMA) guidelines [1,2].

### 4.2. Experimental Design

Table 1 presents our experimental design for the 30-day mortality study on radiation dose effects, and Table 2 presents the biomarker study at early time points (days 3, 7, and 14 post-total-body irradiation).

### 4.3. Total-Body Irradiation

Fifteen minutes prior to total-body irradiation (TBI), all 13–14-week-old mice were placed in ventilated plexiglass containers, with four mice per container. A total of 40 mice were irradiated simultaneously without anesthesia [1,2]. In the RI and RCI groups, radiation was delivered to absorbed doses of 8.5–9.5 Gy at a rate of approximately 0.4 Gy/min from a bilateral radiation field at AFRRI’s 60-Cobalt facility. The dose rate, determined by the distance between the irradiators and animals, was measured using an alanine/electron spin resonance (ESR) dosimetry system with acrylic mouse phantoms (American Society for Testing and Materials, Standard E 1607; ASTI International, Philadelphia, PA, USA) [1,2]. Mice in the sham and wound-alone groups were sham-irradiated and manipulated similarly to those in the RCI group but remained in the cobalt staging room.

### 4.4. Skin Wounding

To prepare for skin wounding, the dorsal fur was shaved using an electric hair clipper under isoflurane anesthesia three days before TBI [1,2]. Within 1–2 h after TBI, a circular-like wound (200–300 mm^2^) was created in the mice from the wound-alone and RCI groups under isoflurane anesthesia, using a 70% ethanol-sterilized steel punch on the skin fold between the shoulder blades [1,2]. After wounding, the mice were placed in autoclaved clean cages with ALPHA-dri bedding (Product# ALPHADRI, manufactured by Shepherd, Kalamazoo, MI, USA, and distributed by Lab Supply, Fort Worth, TX, USA), and the wounds were left open to the environment. All mice subjected to skin injury were then intraperitoneally (i.p.) administered 0.5 mL of acetaminophen solution (150 mg/kg in saline; Acetaminophen injection: NDC 63323, Fresenius Kabi, Lake Zurich, IL, USA) immediately after injury to alleviate pain [1,2]. As a control, 0.5 mL of saline was also i.p administered to groups that received sham irradiation or TBI alone.

### 4.5. Thirty-Day Survival

Mice in the RI and RCI groups were monitored 2–3 times per day for 30 days by investigators and vivarium staff [1,2]. During the critical period of expected mortality (days 10–20 post-TBI), mice were examined three times daily. Moribund mice were euthanized according to USUHS IACUC-approved scoring criteria for establishing early end points in a mouse TBI model [1,2]. Euthanasia was performed by CO_2_ inhalation, followed by confirmatory cervical dislocation. Time-to-death data were recorded, Kaplan–Meier survival curves were plotted, and survival probabilities were calculated at the day 30 post-TBI time point.

### 4.6. Body Weight and Wound Size Measurement

Body weight was measured on days 0 (immediately after TBI), 1, 3, 7, 10, 14, 17, 21, 24, and 28 post-TBI [1,2]. The percentage of body weight (body weight %) was calculated using the following formula: body weight % = 100% ∗ (body weight on day X/body weight on day 0), where “body weight on day X” refers to measurements taken on days 1, 3, 7, 10, 14, 17, 21, 24, or 28 post-TBI and “body weight on day 0” refers to the basal body weight.

Wound size was measured using a digital caliper on days 1, 3, 7, 10, 14, 17, 21, 24, and 28 post-TBI. The average wound area was calculated using the following formula: wound area = π ∗ (A/2) ∗ (B/2), where A and B represent diameters at right angles to each other [1,2]. The percentage of wound closure (% wound closure) was calculated as % wound closure = 100% ∗ [1 − (wound area on day X/wound area on day 1)]. Here, “wound area on day X” refers to measurements taken on days 3, 7, 10, 14, 17, 21, 24, or 28 post-TBI and “wound area on day 1” refers to the basal wound area. A 100% wound closure indicates a fully closed wound.

### 4.7. Blood and Tissue Collection, Peripheral Blood Cell Count, and Serum Preparation

At specified time points, days 3, 7, and 14 post-9.0 Gy RCI, blood was drawn under anesthesia (3% isoflurane, 97% O_2_) via cardiac puncture. A volume of 30–50 µL of blood was collected into a microtube containing EDTA for peripheral blood cell count, while the remaining blood was collected into a microtube with a serum separator additive for serum preparation [1,2]. Following blood draw, euthanasia was performed via cervical dislocation. Skin tissue around the wounds was then collected and divided into two pieces. One half was fixed in 10% formalin for histological examination, while the other half was snap frozen in liquid nitrogen for enzyme-linked immunosorbent assay (ELISA) analysis.

Whole blood samples in EDTA-containing microtubes were analyzed for complete blood count with differential using a clinical hematoanalyzer (Element HT5; Heska, Loveland, CO, USA) following the manufacturer’s instructions. For blood collected in serum separating tubes, sera were obtained after centrifugation at 10,000× *g* for 10 min, following 30–120 min of coagulation at room temperature [1,2]. Sera were immediately aliquoted and stored at −80 °C for ELISA analysis.

### 4.8. Skin Tissue Lysate Preparation

To prepare the skin tissue lysates, frozen skin tissue was washed in ice-cold PBS and then homogenized in ice-cold PBS containing 10 mM N-Ethylmaleimide (NEM, Cat# E3876, Sigma, Saint Louis, MO, USA) and 1× Halt^TM^ Protease & Phosphatase inhibitor (Cat# 1862495, Thermo Scientific, Rockford, IL, USA) [1]. This was performed using a bead lysis kit (Cat# GREENE5, Next Advance, Troy, NY, USA) and a Bullet Blender (Storm 24, Next Advance, Troy, NY, USA) according to the manufacturer’s instructions. After homogenization, supernatant-1 was collected by centrifugation at 13,000× *g* for 20 min at 4 °C. The remaining pellet was resuspended in ice-cold PBS containing 0.5% (*w*/*v*) Hexadecyltrimethylammonium bromide (HTA-Br, Cat# H5882, Sigma, Saint Louis, MO, USA) and 1× Halt^TM^ Protease & Phosphatase inhibitor, and then homogenized again using the same Bullet Blender [1]. Supernatant-2 collected after this second round of centrifugation was mixed with supernatant-1. The protein concentration in the skin tissue lysate was measured using the Pierce^TM^ BAC protein assay kit (REF# 23225, Thermo Scientific, Rockford, IL, USA) according to the manufacturer’s instructions.

### 4.9. N-Acetylglucosaminidase Activity and Myeloperoxidase Activity Assays

β-NAG activity and MPO activity in the skin tissue lysates were measured using the N-Acetylglucosaminidase Activity Assay Kit (Colorimetric, Cat# ab204705, abcam, Waltham, MA, USA) and the Myeloperoxidase Activity Assay Kit (Colorimetric, Cat# ab105136, abcam, Waltham, MA, USA), respectively, according to the manufacturer’s instructions.

### 4.10. Enzyme-Linked Immunosorbent Assay

Following the manufacturer’s instructions, the serum and/or skin tissue levels of the following factors were determined by ELISA: VEGF, Ang-1, Ang-2, IGF-1, ICAM-1, TNF-α, CXCL1/KC, TGFβ1, TGFβ2, TGFβ3, CTGF, and fibronectin. The kits used were as follows: Mouse VEGF Immunoassay (MMV00-1, R&D Systems, Inc., Minneapolis, MN, USA), Mouse Angiopoietin-1 ELISA Kit (Colorimetric, NBP3-18634, Novus Biologicals, Centennial, CO, USA), Mouse/Rat Angiopoietin-2 Immunoassay (MANG20, R&D Systems, Inc., Minneapolis, MN, USA), Mouse/Rat IGF-I/IGF-1 Immunoassay (MG100, R&D Systems, Inc., Minneapolis, MN, USA), ICAM1 (CD54) Mouse ELISA Kit (ab100688, abcam, Waltham, MA, USA), Mouse TNF-α Immunoassay (MTA00B-1, R&D Systems, Inc., Minneapolis, MN, USA), Mouse CXCL1/KC Immunoassay (MKC00B-1, R&D Systems, Inc., Minneapolis, MN, USA), Bio-Plex Pro^TM^ TGF-β 3-plex assay (171W4001M, Bio-Rad Laboratories, Inc., Hercules, CA, USA), Mouse CTGF ELISA Kit (ab289838, abcam, Waltham, CA, USA), and Mouse Fibronectin ELISA Kit (NBP2-60517, Novus Biologicals, Centennial, CO, USA).

### 4.11. Histological Examination of Skin

Skin tissue around the wounds was immediately fixed in 10% formalin for 24–26 h, washed three times with PBS, and then stored in 70% ethanol at room temperature [1,2]. For histological examination, the tissue was dehydrated, embedded in paraffin, and sectioned into 5 µm thick cross sections [1,2]. The gross morphology of the wounds was assessed using H&E staining [1,2]. To evaluate the presence of the myofibroblasts in the skin wounds, the expression levels of α-SMA, a marker of myofibroblast, were determined by IHC staining on formalin-fixed and paraffin-embedded sections. Briefly, sections were incubated overnight at 4 °C with rabbit anti-α-SMA (1:500 dilution, Cat# 124964, abcam, Waltham, MA, USA). Following extensive washes with PBS, sections were incubated for one hour at RT with Goat anti-Rabbit IgG (H+L) Cross-Adsorbed Secondary Antibody, Alexa Fluor™ 568 (1:200 dilution, Cat# A-11011, Invitrogen, Carlsbad, CA, USA) and mounted with ProLong™ Glass Antifade Mountant with NucBlue™ Stain (Cat# P36981, Invitrogen, Carlsbad, CA, USA) [1]. To detect collagen fibers in the mouse skin, formalin-fixed and paraffin-embedded sections were stained with Masson’s trichrome Stain Kit (Cat# 25088-1, Polysciences, Warrington, PA, USA) following the manufacturer’s instruction, where collagen fibers were stained blue; nuclei, black; and cytoplasm, muscle, and erythrocytes red. Both bright-field and fluorescent images were acquired using the Zeiss Axioscan.Z1 and analyzed with Zeiss Zen 2.5 (blue edition) (Carl Zeiss AG, Oberkochen, Germany) [1].

### 4.12. Statistical Analysis

We employed a probit regression model within IBM SPSS Statistics (Armonk, NY, USA) to evaluate the relationship between radiation dose and mortality probability, yielding LD10/30 to LD90/30 values for both RI and RCI [55]. Other data were analyzed using GraphPad Prism 10 (version 10.2.3, San Diego, CA, USA) [1,2]. For 30-day survival, a Log-rank (Mantel-Cox) test was used to compare Kaplan–Meier survival curves [1,2]. For non-survival data, the results were expressed as mean ± standard error of the mean (SEM). Differences among/between groups were analyzed using either one-way or two-way analysis of variance (ANOVA), followed by Dunnett’s, Sidak’s, or Tukey’s multiple comparisons, or an unpaired two-way *t*-test [1,2]. A *p*-value of less than 0.05 was considered statistically significant.

## Figures and Tables

**Table 1:** 30-day mortality study on radiation dose effects.

	8.5 Gy (#)	8.85 Gy (#)	9.25 Gy (#)	9.5 Gy (#)	Total (#)
RI	20	20	20	20	80
RCI	20	20	20	20	80
Total (#)	40	40	40	40	160

# number of animals used.

**Table 2:** Biomarker study at early time points.

	Sham (#)	Wound (#)	9.0 Gy RCI (#)	Total (#)
Day 3		5	5	10
Day 7		5	5	10
Day 14	5 *	5	10 ^	20
Total (#)	5	15	20	40

# number of animals used. * Sham animals were sampled only on day 14 post-sham irradiation to minimize the number of animals used, as we anticipated minimal variations in physiological parameters among sham animals at these time points (days 3, 7, and 14 post-sham irradiation). ^ On day 14, the 9.0 Gy RCI group consisted of 10 mice to ensure adequate data despite potential losses during the critical period. By day 14, nine RCI mice had survived.

## Data Availability

The data presented in this study are available from the corresponding authors on request.
